# Study of fecal glucocorticoid metabolites in captive Asian elephants in Kanchanaburi Province, Thailand

**DOI:** 10.14202/vetworld.2022.647-654

**Published:** 2022-03-23

**Authors:** Weerapun Nokkaew, Apiradee Intarapuk, Apichaya Sakulthai, Worawidh Wajjwalku, Nikorn Thongtip

**Affiliations:** 1 Graduate Program in Bio-Veterinary Science, Faculty of Veterinary Medicine, Kasetsart University, Chatuchak, Bangkok 10900, Thailand; 2 Wildlife Clinic, Faculty of Veterinary Medicine, Mahanakorn University of Technology, Nong Chok, Bangkok 10530, Thailand; 3 Public Health Clinic, Faculty of Veterinary Medicine, Mahanakorn University of Technology, Nong Chok, Bangkok 10530, Thailand; 4 Department of Farm Technology Management, Faculty of Agro-Industry, Panyapiwat Institute of Management, Nonthaburi, Thailand; 5 Akkhraratchakumari Veterinary College, Walailak University, Thasala, Nakhonsithammarat 80160, Thailand; 6 Faculty of Veterinary Medicine, Kasetsart University, Kamphaeng Saen Campus, Nakhon Pathom 73140, Thailand; 7 Center for Agricultural Biotechnology, Kasetsart University, Kamphaeng Saen Campus, Nakhon Pathom 73140, Thailand; 8 Center of Excellence on Agricultural Biotechnology (AG-BIO/PERDO-CHE), Bangkok, 10900, Thailand

**Keywords:** captive elephant, fecal glucocorticoid, hormone, stress, trekking

## Abstract

**Background and Aim:**

Over the past two decades, the number of elephant camps in Thailand has increased considerably, and captive elephants have become more popular within the tourism industry. Tourist activities involving elephant exhibitions and trekking potentially affect animal health and welfare. This study aimed to investigate the relationships between a novel stress biomarker, fecal glucocorticoid metabolites (fGCM), and various factors (sex, age, weather season, tourist season, and elephant usage patterns), monitoring the fGCM concentration during and after trekking activities ceased.

**Materials and Methods:**

Fecal samples of 20 captive Asian elephants from two camps in Kanchanaburi Province were collected monthly for 1 year. The fGCM concentrations were measured using enzyme immunoassay and evaluated relative to individual demography, season, and tourist trekking activity. The mean differences of fGCMs concentrations were compared by analysis of variance and t-test statistics according to data types with p<0.5.

**Results:**

Significant differences in mean fGCM concentrations were found between age categories (p=0.001), trekking and non-trekking animals (p=0.039), and during and after trekking (p=0.023). The mean fGCM concentration of elephants aged during 0-44 years (136.7 ng/g) was significantly higher than for animals over 44 years old (107.7 ng/g), and the elephant trekking group (144.9 ng/g) was significantly higher than the other group (124.7 ng/g). Within the trekking group, the mean fGCM concentrations gradually declined to 129.13 ng/g within 8 months of trekking cessation.

**Conclusion:**

Elephant’s ages and activities co-influenced the variance of fGCM concentrations. In addition, permanent tourist activity, especially trekking, can increase elephant stress. This study’s findings can be applied to the health status monitoring of captive elephants and result in improved animal welfare.

## Introduction

The Asian elephant (*Elephas maximus*) is an important and recognized Thai national symbol. There are approximately 3500 captive elephants in Thailand, a dramatic decline from the approximately 100,000 individuals captive in the early 1900s [1,2]. Due to this dramatic population decline, they were declared as a protected species under Thailand’s Wild Animal Reservation and Protection Act in 1992 [3]. Captive elephant welfare and quality of life have become issues of interest for the general public. At present, Thailand’s landscape transformation includes vast deforestation [4,5], which has impacted the lifestyles of captive elephants and their handlers [1,6], who have changed their activities in semi-forested areas from the timber industry to tourism. This includes elephants trekking, performing in shows, dancing, and painting [7,8]. Working several hours a day continuously for many years has contributed to chronic stress for individual animals, directly affected their welfare, and negatively impacted their physiology and behavior. The related studies have documented the impact of stressful conditions in some captive animals, including behavioral changes, tension, vigilance, poor physical condition, and increased cortisol hormone levels [9-13]. Although previous studies reported the prevalence of elevated cortisol in captive elephants [7,14-17], and the stress from transportation and captivity may lead to overt disease [18], information of the relationship between stress conditions and elephant involvement in tourism, especially trekking, has been incomplete. In contrast, the protective effect of continuous trekking, *per se*, may not be entirely harmful since daily exercise may improve an animal’s physical condition and physiological profile [8].

Chronic stress has potentially negative effects on health and reproductive efficacy [10], which relate to prolonged periods of elevated glucocorticoid levels. These effects may inhibit protein synthesis, increase energy mobilization, and decrease fitness by causing immunosuppression, tissue atrophy, poor population performance, and disruption of reproductive hormone secretion [19,20]. A group of hormones, including the adrenocorticotropic hormone (ACTH), glucocorticoids, catecholamine, and prolactin, is involved in the stress response. It initiates from the pituitary gland stimulation, releasing ACTH that upregulates the zona fasciculata cells of the adrenal cortex and increases the synthesis and release of cortisol (circulating glucocorticoids) or corticosterone [21,22]. Understanding the mechanism of specific steroid metabolite secretion through the liver, gall bladder, and intestines before accumulation and excretion in feces [23] enables non-invasive glucocorticoid metabolite monitoring, which has recently become a widely used technique. It is a powerful tool for understanding an animal’s endocrine status, and it offers significant advantages over blood sampling for stress status assessment [24-26]. This measurement of physiological stress has a wide array of applications for conservation biology, wildlife management, animal husbandry, behavioral ecology, and biomedicine [27,28].

Due to the physiological relationship between stress and cortisol levels in animals, fecal glucocorticoid metabolites (fGCMs) measurement has proven useful in identifying factors affecting animal well-being in captivity [16,29,30]. Norkaew *et al*. [8] found fGCM evaluation predictive of high metabolic and lipid levels involved in stress conditions in bull Asian elephants in Thailand. These vary according to the elephant’s activities and during the tourist season. Among free-ranging Asian elephants in seasonally dry tropical forests of southern India, lower body condition scores occur in elephants during the dry season compared to the wet season, with a season-dependent relationship to stress status as measured by fGCM [15]. Similarly, non-invasive hormone measurements provided information on the level of stress experienced by African elephants, with monitoring fGCM concentrations considered to help improve elephant health assessment [14].

Recently, the evaluation of captive elephant response to various human-imposed stressors found that fGCM concentrations were lowest in saddle-ridden elephants than those ridden bareback and non-trekking groups [17]. In addition, there were factors such as better welfare, feeding, and increased social time, which restrained the decrease in fGCM concentrations. This study aimed to compare fGCM concentrations of the elephants between both captive Asian elephant camps with tourist activity differences in Kanchanaburi Province and to evaluate the influences of various factors (sex, age, season, season for tourism, and elephant usage patterns) on their fGCM concentrations. These data could be applicable for monitoring the health status of captive elephants and improving elephant camp management. These then promote the physical and mental health of captive elephants.

## Materials and Methods

### Ethical approval

The authors assert that all procedures contributing to this work were complied with the ethical standards and approved by the Institutional Review Board at the Faculty of Veterinary Medicine, Mahanakorn University of Technology (Approval No. ACUC-MUT-2021-004).

### Study period, areas, and experiment animals

The field survey took place from May 2015 to April 2016 in Kanchanaburi Province, Western Thailand, where the geography consists of fertile forests, mountains, and plains around the confluence of the Khwae Noi and Khwae Yai Rivers. The area shares a border with Myanmar and has the Tanaowasi Range as its borderline to the west. The climate is tropical savanna, but it is dry in the winter. The average annual temperature is 29.4°C, with the highest average temperatures occurring in March and April (40.3-41.5°C) and the lowest average temperatures occurring in December (16°C) [31]. There were 12 elephant camps located throughout the area involved in various activities. The selection criteria were based on similar characteristics of habitat and feeding but different elephant activity. Two camps located on the main rivers met the selected criteria. The first camp was Camp No.1 (14.10939N, 99.14560E), located along the Khwae Noi River in Lum Sum sub-district, Sai Yok district. There were 31 elephants (3 males, 28 females, and aged 1-46 years). During 12 h of daytime activity, they performed in shows twice a day and conducted 20-min long tourist trekking rides within and surrounding the camp between 09:30 and 15:00. They were individually restrained for 2-3 h while not working. When trekking activities ceased in August 2015, alternative activities included relaxing walks in green fields, roaming free, playing in the mud, eating supplementary foods, and swimming in the river. The second camp was Camp No.2 (14.134863N, 99.322924E) located along the Khwae Yai River in Wang Dong sub-district, Mueang-Kanchanaburi district. There were 20 elephants (4 males, 16 females, and aged 2-76 years) that did not participate in shows or trekking and only had activities involving tourists during feeding and daily bathing. All the elephants were under individual mahout care 24 h daily at both camps. They were individually fed between 18:00 and 06:00 h (Table-1), and tethered for 12 h with a 5-m long chain in a separate camp area. During the 12 h of daytime activity, they were restrained for 2-3 h during the morning session of feeding (Table-1). Primary healthcare was provided by a veterinarian semiannually. This study randomly selected ten healthy elephants per camp, excluding pregnant and musth animals. Ten females aged 16-40 years came from Camp No. 1, and seven females and three males aged 4-76 years came from Camp No. 2. They were divided into two age groups: Working elephants aged 0-44 years; and those over 44 years old, including retired elephants [12].

**Table-1:** Description of the activities and diet of elephants that participated in this study.

	Camp No. 1	Camp No. 2
Activity		
Daytime	Performing show (2 times/day)	Take the mud spa
	Elephant trekking	Feeding by tourist
	Feeding by tourist	Bathe elephant
	Bathe elephant	
Nighttime	Separated tethering (12 h)	Separated tethering
Trekking		
Round time (min)	20 min	0
Trekking time	1 up to 2 h/day	0
Diet		
Daytime	Main diet	Main diet
	Pineapple tree	Pineapple tree
	Supplement diet	Supplement diet
	Banana	Banana
	Banana grass and tree	Banana grass and tree
	Sugarcane	Sugarcane
		Seasonal fruits and vegetables
	Water *ad libitum* from the river in the morning, afternoon and evening	Water *ad libitum* from the river in the morning, afternoon and evening
Nighttime	Main diet	Main diet
	Pineapple tree	Pineapple tree

### Seasonal determination

The three major seasons of Thailand are summer (mid-February to mid-May), rainy (mid-May to mid-October), and winter (mid-October to mid-February). Kanchanaburi can be visited throughout the year, but the peak months for tourism are from November through February (high season) when the temperature is cooler. Information on daily temperature (°C) and rainfall (mm) during the study period at each facility was obtained from the Climatological Center, Meteorological Department of Thailand [31].

### Sampling period and fecal collection

Fecal samples from the 20 elephants selected from both camps were collected during the 1^st^ week of each month from May 2015 to April 2016. About 50 g of fresh fecal samples were collected between 07:00 and 08:30. The well-mixed samples were placed in zip-locked plastic bags, stored in an icebox for transportation (1-2 h), and kept in a −20°C refrigerator in the laboratory pending further extraction and analysis.

### Fecal extraction

Frozen fecal samples were dried in a hot air oven at 60°C for approximately 72 h then cooled to room temperature (RT). Dried feces were immediately extracted with ethanol following the procedure described by Brown *et al*. [32]. Briefly, 0.1 g dried fecal samples were added to 5.0 mL of 90% ethanol and boiled at 96°C for 20 min with 100% ethanol added as needed to keep from boiling dry. The samples were centrifuged at 1500 × g for 20 min, and extracts were poured off into the second set of glass tubes. About 90% ethanol was added to the remaining fecal pellets and centrifuged at 1500 × g for 15 min. Then the first and second extracts were combined and dried in a hot air oven at 50°C. One mL methanol was added to the dry sample, and the samples containing steroid metabolites were stored at −20°C pending analysis.

### fGCM analysis using enzyme immunoassay (EIA)

The fecal concentrations of glucocorticoid metabolites were measured using a single antibody competitive EIA with a polyclonal rabbit anti-corticosterone antibody (CJM006; 25 mL, 1:20,000 dilution), which has been validated for Asian elephants [33]. Secondary antibody-coated plates were prepared by adding anti-rabbit IgG (0.01 mg/mL, 1:60,000) to each well of a 96-well Nunc-Immuno MaxiSorp microtiter plate and incubating at RT for 24 h. The wells were emptied and blotted dry, then added blocking solution and incubated for 15-24 h at RT. After incubation, all wells were emptied, blotted, and dried at RT with loose desiccant in the bottom. After drying (humidity <20%), plates were heat-sealed in a foil bag with a desiccant packet and stored at 4°C until used. Samples diluted at 1:1 in the assay buffer and corticosterone standards (3.9-1000 pg/well; Sigma Diagnostics, St. Louis, MO) were added to appropriate wells. The horseradish peroxidase-conjugated antibody (1:100) was labeled corticosterone (Coralie Munro, University of California, Davis, USA) and used with the microtiter plate reader for quantitative corticosterone detection (405 nm). The intra- and inter-assay coefficients of variation were 5.32% and 6.88% (*n* = 185), respectively. Data are expressed as ng/g of dry feces.

### Statistical analysis

Results were expressed as mean±standard deviation (SD) of fGCM concentrations, of which mean differences were evaluated by analysis of variance and t-test. Repeated measurement analyses of variance were performed to study variance at elephant camps over time. Scheffe *post hoc* tests were further used to analyze differences in mean fGCM concentrations between activity groups and months. Independent and paired t-tests were analyzed to compare fGCM concentrations by elephant demography, tourist season, and elephant trekking activity according to types of data. A completely randomized design was used for seasonal analysis. p<0.5 was considered statistically significant. The Statistic Package for the Social Sciences (SPSS 13.0 for Windows, SPSS Inc. Chicago, IL, USA) was used for data analysis.

## Results

**Figure-1:**
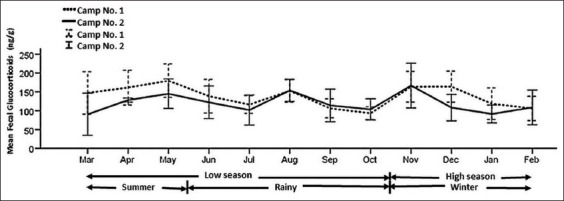
Mean (±standard deviation) fecal glucocorticoid metabolite concentrations of elephants at Camp No. 1 and Camp No. 2 varied by season (summer, rainy, and winter) and tourist season (low and high).

The fGCM concentrations and data from 20 Asian elephants from two study sites were collected monthly for 1 year. Descriptive statistics for fGCM concentrations are presented in Table-2. The ten elephants in Camp No. 1 were young female working elephants with an average age of 26.4 years old. The Camp No. 2 elephants consisted of three males and seven females with an average age of 54.6 years old and included two working and eight retired elephants. The mean (±SD) fGCM concentrations profiled from all 20 elephants year-round were 126.7±46 ng/g. There was a significant difference in the fGCM concentrations between Camp No. 1 (135.2±44.3 ng/g) and Camp No. 2 (116.1±44 ng/g) (p<0.05). There was no difference in fGCM concentrations between the camps by time effect during time interval changes (p>0.05) (Figure-1).

**Table-2:** Comparison among various factors influencing fecal glucocorticoid metabolite concentrations (ng/g) in elephants. Fecal samples had been periodically collected from the same elephants in both camps for 1 year.

Factor	Mean of fecal glucocorticoid metabolite concentrations (ng/g)			Number of elephants	Statistics	p-value

	Camp No. 1	Camp No. 2	Mean hormone (±standard deviation)			
Sex					–1.031	0.312
Male	-	118.2±57.9	118.24±57.9	3		
Female	135.2±44.3	115.3±37.5	127.5±42.9	17		
Age (years)					4.315	0.001*
0-44	135.2±44.3	145.2±57.9^a^	136.7±46.5^a^	11		
>44	-	107.7±35.4^b^	107.7±35.4^b^	9		
Season					2.757	0.066
Summer (Mar-May)	160.8±48.5^a^	115.0±52.0	144.2±53.9	20		
Rainy (Jun-Oct)	122.7±36.1^b^	116.8±40.1	119.9±37.9	20		
Winter (Nov-Feb)	136.7±46.3	115.7±47.5	126.9±47.6	20		
Tourist season					–1.774	0.086
High (Nov-Feb)	136.7±46.3	115.7±47.5	126.9±47.6	20		
Low (Mar-Oct)	134.5±43.7	116.4±42.6	126.6±44.0	20		
Usage patterns (May to September)					–2.11	0.039*
Trekking	144.9±39.6	-	144.9.6±39.6^a^	10		
Non-Trekking	-	124.7±43.1	124.7±43.1^b^	10		
Usage pattern within camp					–2.381	0.023*
Trekking (May-Sep)	144.9.6±6.4^a^	-	144.9.6±6.4^a^	10		
Non-Trekking (Oct-Apr)	129.13±46.3^b^	-	129.13±46.3^b^	10		

^a,b^Values differ among variables, different letters indicate differences.

* Significantly different (p<0.05)

**Figure-2:**
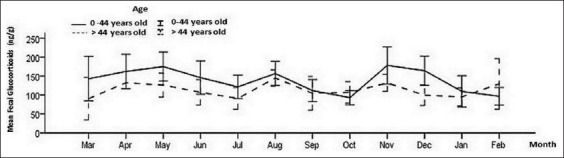
Mean (±standard deviation) fecal glucocorticoid metabolite concentrations of elephants by age class.

The comparison of fGCM concentrations among demographic factors summarized in Table-2 showed that gender did not influence hormone levels (p<0.05). In contrast, the mean of fGCM differed significantly across age classes (p<0.01) and was higher in the class of elephants aged 0-44 years (136.7 ng/g) than in those aged over 44 years (107.7 ng/g) (Figure-2). In Camp No.2, the mean fGCM were significantly different between age classes (p<0.01).

Although time intervals determined by local climate and tourist numbers should affect the stress condition of captive elephants, neither impacted fGCM concentrations. There were no significant differences in the seasonal fGCM means (p>0.05). The peak levels increased during summer (144.2 ng/g), and lowered in winter (126.9 ng/g) and the rainy season (119.9 ng/g). Furthermore, the highest significance between summer and the rainy season was found in Camp No.1 (p<0.05). The most popular tourist season of November to February was compared to other seasons and there was no difference in elephant fGCM concentrations (p>0.05). Because of the limited duration of elephant trekking activities in Camp No.1, fGCM between both camps from May to August. The mean of fGCM (144.9 ng/g) in the elephant trekking group was significantly different from the other group (124.7 ng/g) (p<0.05). The concentrations were compared both during and after the trekking activity to prove that the cessation of trekking resulted in diminished fGCM. There was a significant fGCM decrease after the elephant’s lifestyle had changed (p<0.05).

## Discussion

This study aimed to determine the effect of activities on variations in fGCM concentrations of captive Asian elephants in two different camps with elephant activities (i.e., trekking as a main activity with an average 1-2 h/day) in related to tourist season, season, and demography. The fGCM concentrations could refer to a biomarker of chronic stress in Asian elephants and other species [34,35]. Like cortisol [36], activity-dependent fGCM in normal elephants follow a circadian rhythm. Therefore, the regulation of elephant activity can increase the fGCM concentrations during daytime activities. This study observed fluctuations correlated to carefully controlled conditions between the groups during daytime activities, including limited elephant social time, elephant restraining, mahout exposure as a human preference, and care with sufficient food and supplements. Significant differences in fGCM concentrations between the two camps with different elephant activities and significant decreases in fGCM concentrations after elephant trekking ceased within one group highlighted the effect of tourist activity. Samples were collected from two camps, increasing sample sizes and allowing comparison among types of tourist trekking (saddle, howdah, or bareback riding), and are essential to increasing information for better understanding factors affecting elephant health.

Comparing various fGCM concentrations, this study identified statistical differences as factors of age groups and elephant usage patterns (trekking and non-trekking). The study was specifically divided into two age groups as 0-44 years old and above 44 years old based on the elephant’s activities. The cutoff value was determined between working and retired groups. The group aged over 44 years had a mean age of 63 years and ranged 47-76. It had lower fGCM concentrations resulting from non-active physiological behavior and less routine daytime activity [37,38]. This correlates with data from a study of captive elephants in Chiang Mai, Thailand. There bull elephants aged over 30 years presented significantly lower fGCM than other animals [8] and showed decreases in basal metabolism in elderly animals. In contrast, there was no significant variation in fGCM concentrations across age classifications within groups of the world’s largest semi-captive population of Asian elephants, which are employed in the timber industry in Myanmar [12], nor in free-ranging Asian [15] and African [39,40] elephants. These studies suggested that the elephant’s age and activity co-influenced variation of fGCM concentrations.

Stress hormone concentrations correlated with management and tourist activities, with significant differences being identified between camps when tourist trekking operated continuously in only Camp No. 1. There the mean concentration of fGCM was higher in trekking elephants. The previous study has found that fGCM concentration increases could potentially be associated with timber industry elephants shifting between rest periods and intense work [12]. As measured by salivary cortisol concentration, horse stress levels increased with increasing intensity and duration [41].

The association between activity and fGCM concentrations could be investigated further by measuring activity levels through various means. For example, combined accelerometer data and fGCM concentration data could measure energy usage [12]. In addition, the concentration of cortisol or its metabolites and the expression of certain behaviors have been used as indicators to assess the effect of visitor presence on animal welfare. These effects depended on various factors, including species [9-12,29,42]. Furthermore, individuals of the same species may show very different responses to visitors due to each animal’s temperament, personality, and previous experiences [43].

There were significant differences relating to the effect of trekking when examining trekking and post-trekking periods in Camp No. 1. High fGCM concentrations induced by repetitive stimulating activities gradually declined within 8 months after an elephant had stopped working. A relevant study at the Terra Natura Zoological Park, Alicante, Spain, found salivary cortisol concentrations of Asian elephants and Indian rhinoceros utilized to evaluate the effect of zoo openings were significantly higher during opening periods compared with pre- and post-opening periods [9]. During the opening period was the time being particularly stressful for all animals because of increasing their activities from direct contact with visitors, whereas the cortisol was decreased as their stress conditions were copped in post-opening period. In addition, periodic breaks from work for elephants reflected stress relief and showed recovering from the physical effects of work, especially lesions on the back and tail caused by the movement of saddles and girth straps used in tourist trekking [44].

The mean concentrations of fGCM did not differ between genders, as shown by Norkaew *et al*. [8]. They reported significantly higher fGCM concentrations in captive male elephants relative to females in Chiang Mai. In contrast, a report of a higher mean serum cortisol concentration in Asian elephant females (23.3 ng/dL) compared with males (14.4 ng/dL) has also been documented [45]. The unexpected results may have been due to the limited sample size of elephants in the studies. Several studies reported fGCM concentration differences, including higher concentrations in females of several species, including Asian elephants, African wild dogs, clouded leopards, chimpanzees, and Steller sea lions, or no difference between the sexes (red deer, black, and white rhinoceros) [23,45,46]. Therefore, glucocorticoid levels may be related to various behavioral and physiological changes [23].

The fGCM concentrations were highest in the summer (March to May) and lowest in the rainy season (June to October). There was a similar relationship among Asian elephant calves in northern Thailand [47] and free-ranging African elephants within Kruger National Park [40]. Using similar mechanisms employed by desert mammals such as camels, Asian elephants can cope with high environmental temperatures such as those which occur during the summer. Furthermore, elephant heat loss is likely assisted by ear flapping, decreasing their body temperature [48]. However, elephants have a low surface area to volume ratio and no identified evaporative heat loss mechanism such as panting, so overheating is possible [49]. Although there was no difference in the hormone concentrations of elephants during each season, a comparison of the working elephants in Camp No. 1 found a significant difference between the summer and rainy seasons. The results suggest an increase in physiological stress resulted from a decline in food quantity and quality in the dry season [50]. However, the study showed that external factors such as climate could only influence working elephants which were always stressed and that extrinsic reinforcement factors could effectively stimulate their GCM. In addition, a study that used the percentage of fecal nitrogen as an indicator of nutritional status showed a dramatic increase from the dry to the wet season in African elephants [51,52]. In contrast, a study of bull elephants in northern Thailand reported a significant association between fGCM and season, with the highest concentrations occurring during winter when temperatures and rainfall were lower [8]. This is likely caused by the increased production of glucocorticoids that enhance catabolic function during the winter. This is a response to cold stress caused when more energy is needed to maintain optimum body temperature and ensure survival in cooler temperatures [46].

Approximately, 2300 captive elephants are employed in the tourism industry of Thailand with 135 elephant camps and tourist establishments which vary by size and activity [53]. Some of the documented camp effects may be related to the number of tourists, and the effect of the tourist season could influence the levels of fGCM in captive Asian elephants. Nevertheless, this study did not find any significant fGCM difference between the high and low tourist seasons, possibly because not all elephants perceive human presence as a stressor. Furthermore, metabolism and fGCM concentration changes may be influenced by tourist numbers, nutrition from visitor feeding, or work activity. In addition, elephant work time and walking distance positively affected health status and reduced stress [54,55]. In a related study, the elevated fGCM of timber elephants may have been associated with intense workloads and may not represent chronic stress [12]. In contrast, a significant fGCM effect was associated with the tourist season, with higher concentrations in female elephants during the high tourist season than other times, suggesting some tourist activities compromise the elephants’ welfare and negatively affect their behavior and physiology [2]. A study of African elephants and black rhinoceros at the Brookfield Zoo showed the highest fGCM concentrations in summer and lowest in winter. These were related to visitor attendance numbers at the exhibit, which are highest during the summer [42].

Similarly, Pifarré *et al*. [56] indicated that the number of visitors to a zoo influenced the behavior and adrenal activity of 12 Mexican wolves (a critically endangered native species) in three central Mexican zoos. Finally, further studies should examine differences in activity schedules and visitors after sampling working elephants during high tourist seasons. This is because intense workloads may be a cofactor in the variability of baseline concentrations of fGCM.

## Conclusion

Using fGCM measurements to assess higher metabolic activity could help predict stress conditions in captive Asian elephants. The elevated fGCM found were directly related to the elephants’ age and tourist activity and were most prevalent among young working elephants with vigorous metabolisms. In contrast, weather conditions and tourist season did not relate to fGCM concentrations. The study suggested that permanent work with tourist activity, especially trekking, can inevitably increase elephant stress levels. Therefore, special consideration for these elephants should ensure physical and mental health care, including good management practices, and sufficient rest periods. Furthermore, the study proves that intermittent breaks from work throughout the year can relieve stress and promote elephant health.

## Authors’ Contributions

NT and WW: Conceived the study design. WN: Performed sample collections. WW and AS: Conducted laboratory analyses. AI, NT, and WN: Analyzed and interpreted the data and drafted and revised the manuscript. All authors read and approved the final manuscript.
